# Evaluation of the effect of autophagy on pseudoexfoliation syndrome

**DOI:** 10.1177/03000605241306612

**Published:** 2025-09-09

**Authors:** Nazlı Can, Göksu Alaçamlı, Aylin Karalezli, Nermin Başsimitci

**Affiliations:** 1 Department of Obstetrics and Gynecology, Faculty of Medicine, Mugla Sıtkı Koçman University, Turkey; 2 Department of Ophthalmology, Faculty of Medicine, Mugla University, Turkey; 3 Karelezli Private Ophthalmology Clinic, Turkey; 4 Medicine Faculty, Muğla Sıtkı Koçman University, Turkey

**Keywords:** Pseudoexfoliation syndrome, autophagy, immunohistochemisty

## Abstract

**Objective:**

Pseudoexfoliation syndrome is a systemic disease of unknown etiology, seen in advanced ages, characterized by extracellular material accumulation in ocular tissues and visceral organs. Autophagy, which is a basic metabolic pathway, provides macromolecule recycling of the cell and maintains cell homeostasis by adapting to the cell’s stress environment. The aim of this study was to examine the relationship between specific mechanisms of autophagy and pseudoexfoliation syndrome.

**Methods:**

In this prospective–controlled study, anterior capsulorhexis materials obtained during cataract surgery were immunohistochemically compared between eyes with and without pseudoexfoliation syndrome using Image Tool Software for possible changes in autophagy, in terms of expression levels of Beclin 1, ATG5, ATG6, ATG12, p62, and LC3A/B autophagy markers.

**Results:**

Significant differences were observed between the anterior capsulorhexis materials of eyes with and without pseudoexfoliation syndrome, in terms of expression levels of some specific autophagy markers.

**Conclusion:**

Our study may gain importance as it is the first study in which Beclin 1, ATG5, ATG6, ATG12, p62, and LC3A/B autophagy markers are examined prospectively in the etiopathogenesis of pseudoexfoliation syndrome, comprehensively and with a control group.

## Introduction

Pseudoexfoliation is characterized by extracellular deposition of materials consisting of amyloid, laminin, elastic fibers, collagen, and basement membrane. It was first described by Lindberg in 1917. Pseudoexfoliation syndrome (PES) is diagnosed based on unilateral or bilateral deposition of a white, raised, “dandruff-like” formation in nearly all areas of the human eye, but more importantly in anterior segment structures such as the corneal endothelium, anterior capsule, lens zonules, iris, and trabecular meshwork. The presence of this substance in other organs besides the eyes, such as the heart, lungs, liver, kidneys, cerebral meninges, and blood vessels, indicates that PES is a multiorgan disease.^1–3^

Although the prevalence of PES shows regional and ethnic differences, it increases with aging (0.2%–30% cases over 60 years of age); it is a common condition in older adults worldwide. Race, sex, age, environmental factors (sunlight), and nutritional factors have been shown to be the cause of differences between societies.^4^ PES is involved in the etiology of glaucoma and cataract and increases the risk of intraoperative complications in cataract surgery due to factors such as insufficient dilatation, zonule weakness, and increased fragility of the posterior capsule.^5,6^

Autophagy is a catabolic membrane-trafficking process that leads to sequestration and degradation of intracellular material within lysosomes. It is executed at basal levels in every cell and promotes cellular homeostasis by regulating organelle and protein turnover. In response to various forms of cellular stress, however, the levels and cargo of autophagy can be modulated. In nutrient-deprived states, for example, autophagy can be activated to degrade the cargo for cell-autonomous energy production to promote cell survival. In other contexts, in contrast, autophagy has been shown to contribute to cell death. PES is characterized by abnormal accumulation of protein and cellular material in the eye lens, iris, and other tissues. Oxidative stress plays an important role in PES, as in many age-related diseases. Oxidative stress can trigger autophagy processes. This process helps clear accumulated proteins and damaged organelles. Autophagy occurs more effectively in young cells, whereas this process slows down with aging. When the capacity of cells to clear damaged proteins and organelles through autophagy decreases, this may contribute to the development of age-related diseases such as PES. Impairment of autophagic processes in patients with PES indicates that cells are unable to effectively clear accumulated proteins and damaged organelles. This accumulation triggers the characteristic features of PES in eye tissues, namely, abnormal protein accumulations and dysfunction in cells. With aging, deteriorations in mitochondrial functions are observed. Mitochondrial dysfunction is believed to be involved in PES, and failure to properly clear mitochondria can cause further damage to cells by increasing oxidative stress. Specifically, in PES cases seen in older ages, the decrease in autophagy ability with aging contributes to the pathogenesis of PES by leading to increased protein accumulation and cellular damage. Therefore, regulating autophagy processes and treating autophagy disorders may be an important approach in the treatment of PES, as in other age-related neurodegenerative disorders.^7–10^ The diagram below illustrates autophagy and the PES cycle, which is an age-related neurodegenerative disorder.
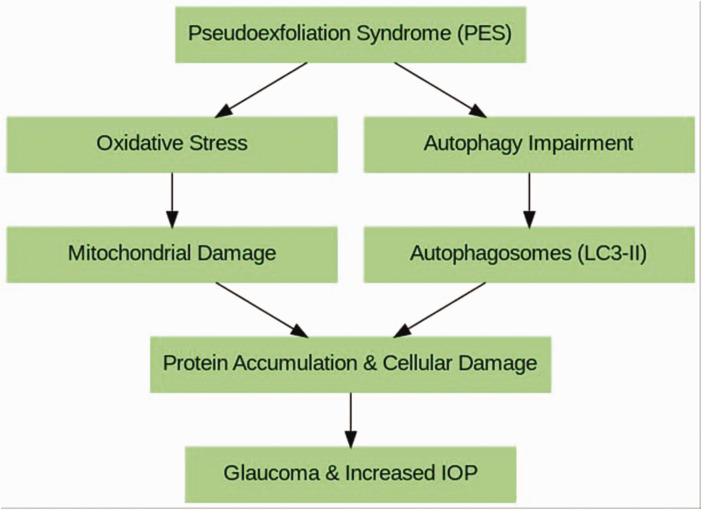


The aim of this study was to evaluate whether the protein expression levels of six key autophagy-related markers (Beclin1, LC3A/B, ATG5, ATG12, ATG16, and p62) are involved in the development of PES.

## Methods

In this study, which was planned prospectively and with a control group, the cases were divided into two groups: PES and control groups. All patients provided written informed consent before participation in the study. Individuals who visited the Ophthalmology outpatient clinic of Mugla University Training and Research Hospital were included in the study.

Patients with clinically significant cataracts and PES constituted the PES group. In contrast, patients with an indication for cataract surgery due to age-related cataract in our outpatient clinic during the same period who did not have PES or any ocular disease constituted the control group. A total of 66 patients were included in the study, with 33 patients in each group. Only single eye of each case was recorded in each group.

Sample size determination and power analysis were performed using G Power software.

Best-corrected visual acuities, biomicroscopic examinations, intraocular pressure measurements, and detailed fundus examinations were performed preoperatively for all cases in both PES and control groups.

Patients who met the following criteria were included in the PES group: (a) in the examination performed after pupil dilation, exfoliation material was seen in the anterior lens capsule and/or pupil edge of the eye to be operated on; (b) intraocular pressure (IOP) of both eyes was below 21 mmHg; and (c) no glaucomatous optic disc changes were required in both eyes.

Patients who met the following criteria were included in the control group: (a) in the examination performed after mydriasis, no exfoliative material was seen in the anterior lens capsule and/or pupil edge of both the eyes; (b) IOP of each eye was below 21 mmHg; and (c) no glaucomatous optic disc changes were required in both eyes.

In the population selected for our study, cases with diabetes mellitus, thyroid dysfunction, liver–kidney failure, and a history of any ocular surgery as well as those who had been diagnosed with glaucoma and refused to participate in the study were excluded from the study. Patients in both groups underwent a routine cataract surgery, during which the anterior capsule material was obtained after the viscoelastic material was injected into the anterior chamber by a successful capsulorhexis. This capsulorhexis material was maintained in 2.5% formaldehyde for immunohistochemical examination. The capsulorhexis material obtained was embedded in paraffin blocks. Subsequently, 0.3-micron thick microtomes were used for cutting paraffin blocks. Samples of the anterior capsule material embedded in paraffin blocks were taken on lysine slides and immunohistochemically stained.

### Immunohistochemical evaluation

Immunohistochemical staining was performed using LC3A/B (Cell Signaling #12741), Beclin 1 (Cell Signaling, CBOW0115051), SQSTM1/p62 D5L7G (Cell Signaling #88588), ATG5 (R&D System Minneapolis, CEDH011712A), ATG12 (Cell Signaling #2010S), and ATG16 (Cell Signaling #8089S) antibodies.

The main goal of immunohistochemical staining is to mark the macromolecules known to be present in the cell and make them visible by staining processes.

After the samples were embedded in paraffin blocks, 0.5-micron sections were taken using a microtome, and the tissues were transferred to lysine slides. The lysine slides were then subjected to dehydration to separate the paraffin in the slides. The tissues on the lysine slides were marked with a pen marker.

Next, 3% H_2_O_2_ was added to the samples, and the samples were kept at room temperature for 10 min. Then, the samples were washed thrice with phosphate-buffered saline (PBS) and dried with blotting paper. Subsequently, the samples were incubated at 37°C for 20 min. The samples were thoroughly washed thrice with PBS and then dried with blotting paper. Peroxidase sequencing solution was dripped onto the samples to be applied to the marked area. After 6 min, the samples were washed with PBS, dried, covered with blocking solution, and kept at room temperature for 30 min. The blocking solution was carefully removed without washing, and the appropriately diluted primary antibody was placed on the samples, which were then incubated overnight at 4°C. The next day, the samples were washed thoroughly with PBS, and biotinylated secondary antibody compatible with the primary antibody was dripped onto them. The samples were incubated in a closed, humid box at room temperature for 30 min. Streptavidin peroxidase conjugate was added to the samples, which were washed thrice with PBS and dried. Then, they were kept at room temperature in a closed, humidified container for 15 min. The 3,3′-diaminobenzidine (DAB) mixture was prepared in accordance with the number of samples, with 27 μL of DAB substrate in 1000 μL of DAB solution. At the end of the incubation period, the DAB mixture was placed on the slides, which were then washed thrice with PBS, and excess water was removed. The slides were kept in this way for 2 min. Then, the samples were washed with distilled water. After Harris hematoxylin was added onto the smear preparations, the base was painted. The smear preparations, which were washed with distilled water, were passed through alcohol and xylene series without drying, and the slide coverslip adhesion was achieved with the help of Entellan liquid.

### H-scoring

Immunohistochemical evaluation was performed for each case in both groups. Ten fields were randomly selected from each sample, positively and negatively stained cells were counted in terms of the antibodies determined in the fields, and the staining ratios were calculated as a percentage. The staining intensity of positively stained cells was evaluated based on three categories as weak (+), moderate (+++), and strong (++++) according to the “H-score method” criteria.

H-score of the patients capsule sample =1 × [% of weak (+) staining cells] + 2 × [% of moderate (+++) staining cells] + 3 × [% of strong (++++) staining cells].

### Statistical analyses

Statistical analyses were performed using Statistical Package for Social Sciences 20 package program. In the evaluation of parametric data, morphometric measurements were performed using UTHSCSA Image Tool 12.00 program. Regarding the statistical analysis of the data, independent t-test was used in the comparison of paired groups, and chi-square test was used in the comparison of qualitative data. Moreover, *p-*values <0.05 were considered to indicate statistical significance.

Our study was approved by the Mugla University Clinical Research Ethics Committee, dated 17/02/2021 and numbered 4/V. The study was conducted in accordance with the Helsinki Declaration of 1975, as revised in 2013. We have de-identified all patient details. The reporting of this study conforms to Strengthening the Reporting of Observational Studies in Epidemiology (STROBE) guidelines.^11^

## Results

Of the 66 patients included in the study, 27 were females and 39 were males. Sex distribution according to the groups was as follows. Of the 33 patients in the control group, 12 (36.3%) were females and 21 (63.7%) were males. Of the 33 patients in the PES group, 10 (30.3%) were females and 23 (70%) were males. There was no statistically significant difference between the two groups in terms of sex (*p* > 0.05). The data related to the sex charts of the patients included in the study are given in Table 1.

**Table 1.** Patients included in the study according to sex and age.

Group	Control (mean ± SD)	PES (mean ± SD)	*p* value
Female	12 (36.3%)	10 (30.3%)	>0.05
Male	21 (63.7%)	23 (69.7%)	>0.05
Age (years)	60.27 ± 6.2747–71 (min–max)	62.96 ± 6.5649–72 (min–max)	0.87

PES: pseudoexfoliation syndrome.

The mean age of the patients in the PES group was 62.96 (range: 47–71) years. In the control group, the mean age of the patients was 60.27 (range: 49–72) years. There was no significant difference between the two groups in terms of age (*p* = 0.87). The ages of the patients included in the study are given in Table 1.

Each tissue obtained for the study was examined histologically and stained using immunohistochemical protocols. The obtained samples were visualized under a light microscope, and H-scoring was performed. The data obtained are shown in Table 2.

**Table 2.** H-scoring results in terms of groups.

Antibody/Group	Control (mean ± SD)	PES (mean ± SD)	*p* value
Beclin 1	64.09 ± 3.63	74.12 ± 14.33	0.000
LC3A/B	77.48 ± 4.73	81.00 ± 6.47	0.014
ATG5	44.45 ± 2.82	44.87 ± 2.92	0.551
ATG12	46.36 ± 3.33	46.90 ± 2.44	0.451
ATG16	44.48 ± 3.04	45.18 ± 3.62	0.401
p62	62.39 ± 3.87	58.00 ± 4.02	0.000

PES: pseudoexfoliation syndrome.

The data obtained after H-scoring between the PES and control groups were statistically analyzed.

Beclin 1 antibody H-scores were 64.09 ± 3.63 in the control group and 74.12 ± 14.33 in the PES group. Beclin 1 expression levels in the PES group were significantly higher than those in the control group (*p* = 0.000).

LC3A/B antibody H-scores were 77.48 ± 4.73 in the control group and 81.00 ± 6.47 in the PES group. LC3A/B expression levels in the PES group were significantly higher than those in the control group (*p* = 0.014).

ATG5 antibody H-scores were 44.45 ± 2.82 in the control group and 44.87 ± 2.92 in the PES group. No significant difference was observed in ATG5 expression levels between the PES and control groups (*p* = 0.551).

ATG12 antibody H-scores were 46.36 ± 3.33 in the control group and 46.90 ± 2.44 in the PES group. No significant difference was observed in ATG12 expression levels between the control and PES groups (*p* = 0.451).

ATG16 antibody H-scores were 44.48 ± 3.04 in the control group and 45.18 ± 3.62 in the PES group. ATG16 expression levels in the PES group were not significantly different from those in the control group (*p* = 0.401).

p62 antibody H-scores were 62.39 ± 3.87 in the control group and 58.00 ± 4.02 in the PES group. The p62 expression levels in the control group were significantly higher than those in the PES group (*p* = 0.000).

**Figure 1.**
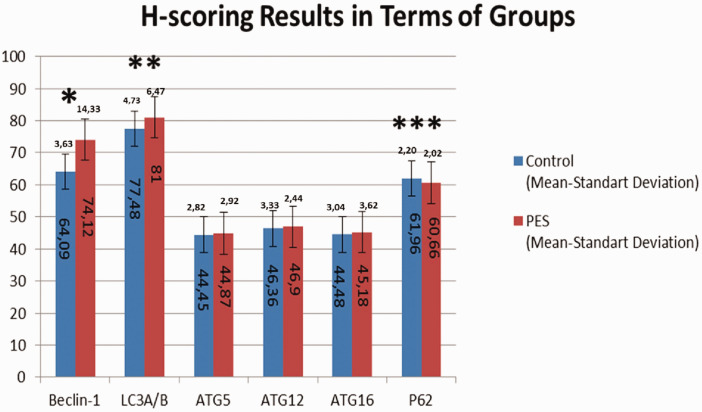
H-scoring results in terms of groups with colored columns. * Presence of statistical significance in Beclin 1 H-scores between the PES group and control group (*p* = 0.000). ** Presence of statistical significance in LC3A/B H-scores between the PES group and control group (*p* = 0.014). *** Presence of statistical significance in p62 H-scores between the PES group and control group (*p* = 0.000). PES: pseudoexfoliation syndrome.

H-scoring results in terms of groups with colored columns are shown in Figure 1.

**Figure 2.**
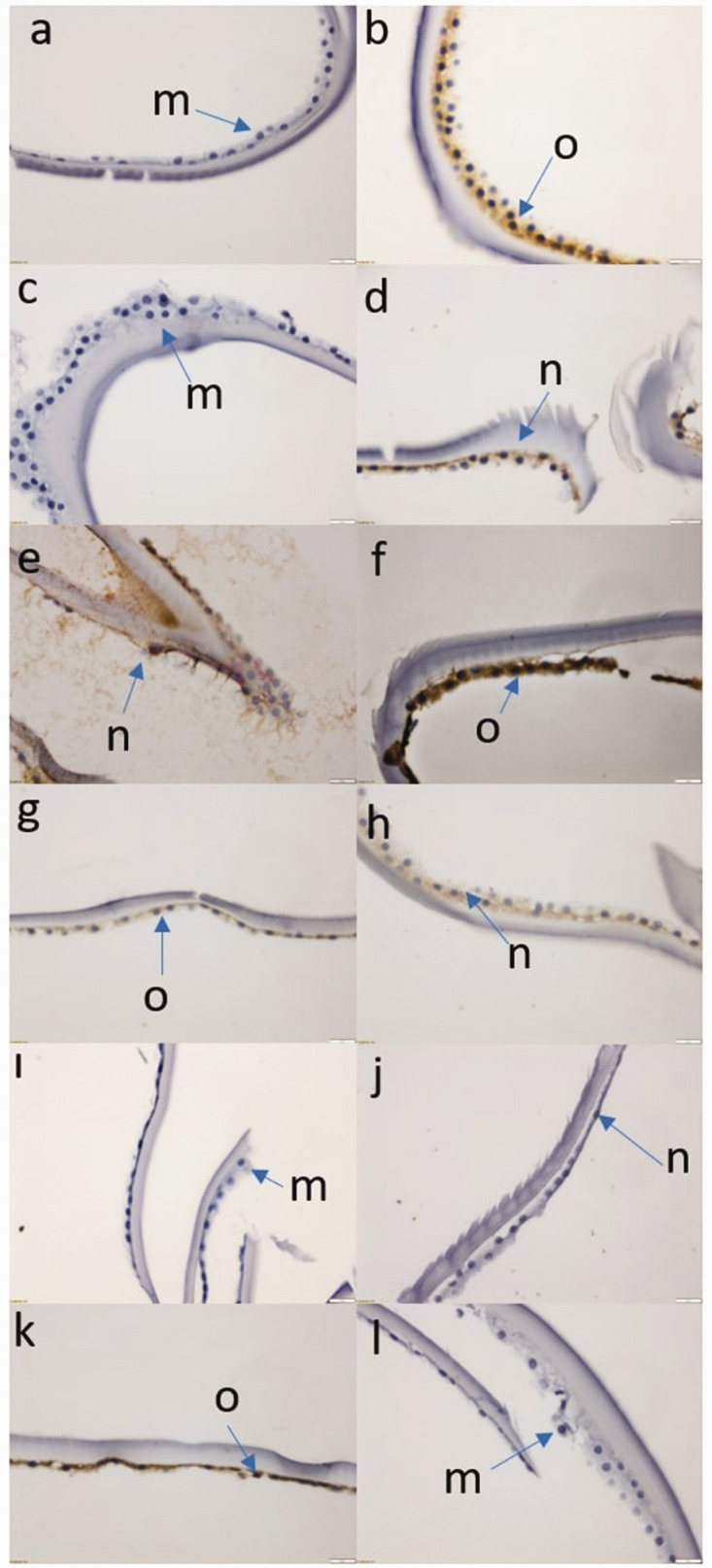
Light microscopic image obtained after immunohistochemical staining (40×). (a) Cell staining in the control group in which Beclin1 was studied, (b) Cell staining in the PES group in which Beclin1 was studied, (c) Cell staining in the control group in which LC3A/B was studied, (d) Cell staining in the PES group in which LC3A/B was studied, (e) Cell staining in the control group in which ATG5 was studied, (f) Cell staining in the PES group in which ATG5 was studied, (g) Cell staining in the control group in which ATG12 was studied, (h) Cell staining in the PES group in which ATG12 was studied, (i) Cell staining in the control group in which ATG16 was studied, (j) Cell staining in the PES group in which ATG16 was studied, (k) Cell staining in the control group in which p62 was studied, (l) Cell staining in the PES group in which p62 was studied, (m) Low (++) staining cells, (n) Middle (+++) staining cells and (o) High (++++) staining cells. PES: pseudoexfoliation syndrome.

Light microscopic images obtained with Beclin1, LC3A/B, ATG5, ATG12, ATG16, and p62 antibodies are displayed in Figure 2.

## Discussion

The aim of this study was to evaluate whether the expression levels of six critical autophagy genes (Beclin1, LC3A/B, ATG5, ATG12, ATG16, and p62) are involved in the development of PES.

PES is an age-related, generalized disorder of the extracellular matrix characterized by the progressive accumulation of abnormal fibrillar material in intra- and extra-ocular tissues. It is estimated to affect approximately 80 million people worldwide and is the second leading cause of blindness.^1–6^ As the pathogenesis of PES has not yet been fully elucidated, many researchers are involved in determining the pathogenesis of PES, and there has been an increase in studies investigating the relationship between PES and autophagy recently. The roles of PES and autophagy have become an important research area. Autophagy is a process by which cells break down and recycle their own components through lysosomes, supporting cellular homeostasis and survival. Clearing intracellular waste and damaged organelles is critical for protecting cellular functions and maintaining cellular health under stress.^7,12^ The accumulation of abnormal fibrils and protein aggregates in PES causes oxidative stress and endoplasmic reticulum stress in cells. Oxidative stress is known as a factor that triggers autophagy and can negatively impact cellular health by causing the accumulation of protein aggregates and toxic substances. Autophagy also plays a role in the regulation of proteins in the extracellular matrix.^9,10^ In PES patients, abnormal accumulation of extracellular matrix contributes to the formation of PES material in the eye. Insufficiency in autophagy mechanisms may prevent the clearance of this material and disrupt the healthy structure of the intercellular matrix.^7–9^ Autophagy plays a critical role in eliminating damaged mitochondria. Mitochondrial dysfunction is common in PES, and this may be associated with autophagy deficiency. Failure to clear damaged mitochondria leads to increased oxidative stress and cellular damage. Studies conducted in recent years have shown that some genes involved in the pathogenesis of PES may be linked to autophagy processes.^10^ For example, the LOXL1 gene is a critical risk factor in the development of PES, and mutations or polymorphisms in this gene can affect autophagy-related processes.^12^ In PES patients, abnormal accumulation of elastic fibers and fibrillar material is characteristic in the intraocular tissues and lens capsule.^9,10,12^ Studies have shown the association of single-nucleotide polymorphisms in *LOXL1*, especially rs1048661 and rs3825942, with this syndrome. However, the exact mechanisms of *LOXL1* variants causing PES pathogenesis are not fully understood. The relationship between *LOXL1* and autophagy can also be considered in the context of cellular stress responses and extracellular matrix regulation. The LOXL1 enzyme plays a critical role in maintaining homeostasis of elastic fibers. In PES, this homeostasis is disrupted, and abnormal protein accumulation occurs. These accumulations can trigger autophagic activity within the cell. If autophagy cannot clear these abnormal proteins and fibrillar materials, this can lead to increased cellular stress.^13,14^ Cell culture studies on PES have attempted to disrupt the autophagic axis by simply withdrawing serum from the media (by creating starvation), and disruptions in microtubule structures were observed.^13^ The observed disruptions in autophagy occurred in two ways. First, there was an increase in the accumulation of LC3-II, a known autophagosome marker. Second, the impairment of the autophagic axis was observed due to the inability to clear the increased autophagosomes in the environment. Considering the results of the study, changes in autophagic mechanisms were observed in PES tissues, as observed in many neurodegenerative diseases. When the results obtained in a study on aging and neurodegenerative diseases involving PES tissue were assessed, changes in the autophagy mechanism, characterized by impaired LC3 expression, swollen and misplaced lysosomes and endosomes, and impaired mitochondria structures, were observed in the ultrastructural examination of PES tissue samples.^9^

Cellular stress conditions are important mechanisms underlying the pathogenesis of PES. One of the potential cellular responses to these stress conditions is the induction of autophagy.^7–9^

Reactive oxygen species (ROS) are associated with the pathophysiological parainflammation and autophagy process in the course of age-related macular degeneration. ROS promote apoptosis of vascular and neuronal cells and stimulate inflammation and pathological angiogenesis in the course of diabetic retinopathy. Superoxide and hydrogen peroxide play a dual role in signaling the autophagy process. Through influence on BECN1 (Beclin 1, autophagy-related), ROS induce the formation of class III phosphatidylinositol 3-kinase (PtdIns3K) complexes and positively regulate autophagy; PtdIns3K is an intracellular energy sensor, which specifically responds to energy depletion.^15–17^ In our study, we found that Beclin 1 expression was significantly higher in the PES group than in the control group.

ROS are involved in the mechanism through ATG4 protein, which plays an important role in autophagosome formation. ATG4 protein plays a role in the formation of LC3 protein in the appropriate and mature form. LC3 is a critical component for autophagosome development and microtubule-dependent migration toward the cell center. The free cytosolic isoform (LC3-I) is converted to an isoform (LC3-II), which binds to developing autophagosomes via lipidation and helps the maturing autophagosome bind to microtubules.^13^ Both LC3I and LC3II are used as markers of autophagy at different stages that reveal the balance of biogenesis and degradation.^14^ ROS are signaling molecules that regulate cell death or survival at various levels in conjunction with autophagy. In our study, although we observed a significant increase in the expression level of Beclin1 biomarker, we did not observe the same increase in the expression levels of ATG5, ATG12, and ATG16 biomarkers. The change in Beclin1 expression level in the autophagy process plays an important role, especially during the initiation of autophagy. In contrast, the levels of other autophagy markers, such as ATG proteins (e.g. ATG5, ATG6, and ATG12), remain constant, owing to the different roles and regulatory mechanisms of these proteins in the autophagy process.^13^ Beclin1 is a critical regulator for the initiation of autophagy. In the early stages of autophagy, it initiates the membrane formation of autophagosomes. When cells are under stress (e.g. nutrient deficiency or oxidative stress), there is an increase in the expression and activity of Beclin1. This increase accelerates the initiation of the autophagy process and the formation of autophagosomes. Therefore, an increase or change in Beclin1 expression levels can be observed in response to autophagy activity.^13^

ATG proteins (e.g. ATG5, ATG6, and ATG12) are involved in different stages of the autophagy process and are required for the maturation and elongation of autophagosomes. ATG proteins function in conjugation systems that support the expansion and closure of autophagosome membranes during the middle stages of autophagy. However, the levels of ATG proteins generally remain constant and do not show large fluctuations, as observed in Beclin1, during autophagy induction. Maintaining ATG proteins at constant levels ensures the regular and continuous functioning of the autophagy process. Beclin1 and ATG proteins involved in different stages of autophagy are subject to different regulatory mechanisms. Changes in the level of Beclin1 are generally controlled by transcriptional and post-translational modifications (phosphorylation and ubiquitination). Conversely, ATG proteins are generally less subject to such regulation, and their intracellular levels remain more stable. Beclin1 is a critical factor that regulates the onset of autophagy; therefore, its levels can be increased in response to cellular stress. However, ATG proteins function in the middle and final stages of autophagy. The amount of protein required at these stages is not as dynamic as at the beginning. Therefore, ATG proteins may remain at a more constant level.^8–10,12,13^

During the induction of autophagy, when proteins such as Beclin1 are required, their expression can be increased; however, as ATG proteins are already present at a certain level, additional expression or an increase in the protein level is usually not required. This can help cells save energy and prevent unnecessary protein production. In summary, the different functions and regulatory patterns of Beclin1 and ATG proteins in autophagy explain why their cellular levels respond differently. Beclin1 levels increase during the induction of autophagy, while ATG proteins generally remain constant because they are already sufficient for their current function and are controlled mostly by post-translational regulatory mechanisms. Therefore, the autophagy process is dynamically regulated.^15–17^

In a previous study, when PES tissue cells were compared with control tissue cells, LC3II accumulation in PES stood out with a yellow color under a fluorescence microscope, while LC3A/B markers were observed to be intensely stained positively in both immunohistochemical examination and the western blot method.^16^ After immunohistochemical staining in our study, we observed a statistically significant increase in the expression of LC3A/B marker. The results obtained in our study were compatible with those of other studies.^15^

Our study is one of the most comprehensive studies on autophagy-related genes with the widest range of autophagy markers. In our study, when we examined the p62 marker levels in the PES tissue, which have never been examined in the literature previously, we observed that the p62 level decreased significantly in the PES tissue.

Our data showed that disorders related to autophagy pathways may contribute to the dysfunction of degradative organelles functioning within the cell, and the resulting accumulation of dysfunctional mitochondria and their interaction with microtubule dynamics may contribute to PES pathology. These data are consistent with those from other studies supporting that autophagy impairment in lysosomal disease results in increased oxidative stress and inactive accumulation of mitochondria.^16,17^

LC3 is a critical marker of the autophagy process and is involved in the formation of autophagosomes. Autophagy is an important cellular process for clearing damaged proteins and organelles in cells.^10,12–14^ Abnormal protein accumulation and oxidative stress in PES may necessitate autophagy in the cell, resulting in an increase in LC3 level. When autophagic flux is impaired, early stages of autophagy can be induced (e.g. LC3-II accumulation); however, autophagosomes may be prevented from fusing with lysosomes and degrading their contents. In this case, the increase in LC3-II levels indicates obstruction or impairment of autophagic flux.^10,12–14^ PES is also associated with mitochondrial dysfunction and decreased energy production. Mitochondrial stress induces autophagy (specifically mitophagy) and causes LC3-II accumulation in this process. Mitochondrial dysfunction requires cells to clear damaged mitochondria through autophagy, which can lead to increased levels of LC3. In our study, we observed an increase in LC3 levels.

Similar to age-related neurodegenerative diseases, decreased p62 levels in PES may be an indicator of changes in the autophagy process.^13–14^ p62 is an adapter protein that is recognized by autophagosomes and degraded via lysosomal degradation. If autophagy activity increases, further degradation of p62 within autophagosomes occurs. In this case, the levels of p62 protein may decrease as a result of increased autophagy activity in patients with PES. Decreased p62 levels indicate that cells use autophagy more actively during stressful conditions or in an effort to clear damaged proteins.

In a state where autophagic flux is functioning properly, all stages of autophagy (autophagosome formation, autophagosome–lysosome fusion, and content degradation) are effectively completed. In this process, the contents of autophagosomes, including p62 protein, are destroyed by lysosomes. If autophagic flux is functioning properly in PES, its levels may decrease due to efficient degradation of p62 by lysosomal degradation. One of the characteristic features observed in patients with PES is abnormal protein and extracellular matrix accumulation. p62 acts as an adapter protein that recognizes and transports such protein aggregates to autophagosomes. When autophagy activity increases, p62 protein is used to clear these aggregates and is subsequently degraded. Increased autophagy activity leads to depletion of p62 protein, thereby decreasing its levels within the cell. The decrease in p62 levels may also be due to the regulatory mechanisms in the early stages of autophagy.^18–19^

Autophagy involves a series of complex regulatory processes, from autophagy initiation to autophagosome formation and lysosomal degradation. Appropriate progress of these processes may result in decreased p62 levels, a clear indicator of autophagy activity. This indicates that cells effectively use autophagy to clear more proteins and organelles in PES. If the activity of lysosomes increases, the contents of autophagosomes (including p62) are degraded more efficiently. Increased lysosomal activity in patients with PES may cause cells to clear more waste material, resulting in decreased p62 levels. Decreased p62 levels in PES generally indicate increased autophagy activity and that cells are maintaining autophagic flux efficiently. As p62 is a critical adapter protein in the clearance of cellular wastes through autophagy, the decrease in its levels may be an indicator of the effectiveness of autophagic processes in patients with PES. However, more research is needed to understand the exact cause and biological consequences of changes in p62 levels.^19–21^

Our results reveal a link between PES pathology and autophagy dysfunction systemically in the body, brain, and retina, which is an important contributor to many age-related diseases. The decrease or disappearance of denatured protein and the degradation capacity of aging cellular organelles may underlie the development of extracellular protein aggregates in PES.

However, it is not possible to determine the genetic variant only by immunohistochemical study. If an evaluation is to be made based on the genetic variant, as stated in the purpose, the DNA sequence must be examined through sequence analysis. This is one of the main limitations of the study.

If the aqueous humor expression levels of these genes at the RNA level had been evaluated, it would be valuable in terms of the power of the study. This is another limitation of the study.

Our findings indicate that different pathways are active in the autophagy mechanism in PES cases with cataract. The close relationship between the autophagy mechanism and tumor formation has been the subject of many studies. It seems possible to develop new treatment strategies in cancer treatment through autophagy genes. In this respect, we believe that the results of our study are extremely valuable, and it would be useful to confirm these results through studies involving a larger number of patients and techniques such as real-time polymerase chain reaction.

## Data Availability

Data are available within the article or its supplementary materials.

## References

[1] Erkayhan GE Dogan S. Cataract surgery and possible complications in patients with pseudoexfoliation syndrome. Eurasian J Med 2017; 49: 22–25. 28416927 10.5152/eurasianjmed.2016.0060 PMC5389488

[2] Parekh P Green WR Stark WJ , et al. Electron microscopic investigation of the lens capsule and conjunctival tissues in individuals with clinically unilateral pseudoexfoliation syndrome. Ophthalmology 2008; 115: 614–619. e2. 17698197 10.1016/j.ophtha.2007.05.039

[3] Schlotzer-Schrehardt UM Koca MR Naumann GO , et al. Pseudoexfoliation syndrome. Ocular manifestation of a systemic disorder? Arch Ophthalmol 1992; 110: 1752–1756. 1463418 10.1001/archopht.1992.01080240092038

[4] Mitchell P Wang JJ Smith W. Association of pseudoexfoliation syndrome with increased vascular risk. Am J Ophthalmol 1997; 124: 685–687. 9372724 10.1016/s0002-9394(14)70908-0

[5] You QS Xu L Wang YX , et al. Pseudoexfoliation: normative data and associations: the Beijing eye study 2011. Ophthalmology 2013; 120: 1551–1558. 23622877 10.1016/j.ophtha.2013.01.020

[6] Fontana L Coassin M Iovieno A , et al. Cataract surgery in patients with pseudoex-foliation syndrome: current updates. Clin Ophthalmol 2017; 11: 1377–1383. 28814824 10.2147/OPTH.S142870 PMC5546806

[7] Wolosin JM Ritch R Bernstein AM. Is autophagy dysfunction a key to exfoliation glaucoma? J Glaucoma 2018; 27: 197–201. 27977481 10.1097/IJG.0000000000000606 PMC5468508

[8] Want A Gillespie SR Wang Z , et al. Autophagy and mitochondrial dysfunction in tenon fibroblasts from exfoliation glaucoma patients. PLoS One 2016; 11: e0157404. 27391778 10.1371/journal.pone.0157404 PMC4938507

[9] Bernstein AM Ritch R Wolosinc JM. LOXL1 folding in exfoliation glaucoma. Adv Protein Chem Struct Biol 2019; 118: 273–288. 31928728 10.1016/bs.apcsb.2019.09.005 PMC7589528

[10] Mauvezin C Neufeld TP. Bafilomycin A1 disrupts autophagic flux by inhibiting both V-ATPase-dependent acidification and Ca-P60A/SERCA-dependent autophagosome-lysosome fusion. Autophagy 2015; 11: 1437–1438. 26156798 10.1080/15548627.2015.1066957 PMC4590655

[11] Von Elm E Altman DG Egger M ; STROBE Initiative et al. The Strengthening the Reporting of Observational Studies in Epidemiology (STROBE) statement: guidelines for reporting observational studies. Ann Intern Med 2007; 147: 573–577. 17938396 10.7326/0003-4819-147-8-200710160-00010

[12] Bernstein AM Ritch R Wolosin JM. Exfoliation syndrome: a disease of autophagy and LOXL1 proteopathy. J Glaucoma 2018; 27: S44–S53. 29547474 10.1097/IJG.0000000000000919 PMC6028293

[13] Chakraborthy M Rao A. A feedback loop between TGF-beta1 and ATG5 mediated by miR-122-5p regulates fibrosis and EMT in human trabecular meshwork cells. Curr Issues Mol Biol 2023; 45: 2381–2392. 36975524 10.3390/cimb45030154 PMC10047315

[14] Yaz Y Yıldırım N Aydın Yaz Y , et al. Three single nucleotide polymorphisms of LOXL1' in a Turkish population with pseudoexfoliation syndrome and pseudoexfoliation glaucoma. Turk J Ophthalmol 2018; 48: 215–220. 30405941 10.4274/tjo.83797 PMC6216538

[15] Tanida I Ueno T Kominami E. LC3 conjugation system in mammalian autophagy. Int J Biochem Cell Biol 2004; 36: 2503–2518. 15325588 10.1016/j.biocel.2004.05.009 PMC7129593

[16] Cao QH Liu F Yang ZL , et al. Prognostic value of autophagy related proteins ULK1, Beclin 1, ATG3, ATG5, ATG7, ATG9, ATG10, ATG12, LC3B and p62/SQSTM1 in gastric cancer. Am J Transl Res 2016; 8: 3831–3847. 27725863 PMC5040681

[17] Nita M Grzybowski A. The role of the reactive oxygen species and oxidative stress in the pathomechanism of the age-related ocular diseases and other pathologies of the anterior and posterior eye segments in adults. Oxid Med Cell Longev 2016; 2016: 3164734. 26881021 10.1155/2016/3164734 PMC4736974

[18] Dong W Cui MC Hu WZ , et al. Evaluation of SQSTM1/p62 on the neuropathologies of Alzheimer’s disease. Front Aging Neurosci 2022; 14: 829232. 35296031 10.3389/fnagi.2022.829232 PMC8919032

[19] Salminen A Kaarniranta K Haapasalo A , et al. Emerging role of p62/sequestosome-1 in the pathogenesis of Alzheimer's disease. Prog Neurobiol 2012; 96: 87–95. 22138392 10.1016/j.pneurobio.2011.11.005

[20] Wang X Jia J. Magnolol improves Alzheimer's disease-like pathologies and cognitive decline by promoting autophagy through activation of the AMPK/mTOR/ULK1 pathway. Biomed Pharmacother 2023; 161: 114473. 36889111 10.1016/j.biopha.2023.114473

[21] Guillot-Sestier MV Sunyach C Druon C , et al. The alpha-secretase-derived N-terminal product of cellular prion, N1, displays neuroprotective function in vitro and in vivo. J Biol Chem 2009; 284: 35973–35986. 19850936 10.1074/jbc.M109.051086 PMC2791025

